# Longitudinal metagenomic study reveals the dynamics of fecal antibiotic resistome in pigs throughout the lifetime

**DOI:** 10.1186/s42523-023-00279-z

**Published:** 2023-11-08

**Authors:** Lingyan Ma, Yuanyuan Song, Wentao Lyu, Qu Chen, Xingning Xiao, Yuanxiang Jin, Hua Yang, Wen Wang, Yingping Xiao

**Affiliations:** 1 State Key Laboratory for Managing Biotic and Chemical Threats to the Quality and Safety of Agro-products, Institute of Agro-product Safety and Nutrition, Zhejiang Academy of Agricultural Sciences, Hangzhou, 310021 China; 2 College of Biotechnology and Bioengineering, Zhejiang University of Technology, Hangzhou, 310032 China

**Keywords:** Antibiotic resistance genes, Transmission, Gut microbiota, Health risk, Growth stage, Pigs

## Abstract

**Background:**

The dissemination of antibiotic resistance genes (ARGs) poses a substantial threat to environmental safety and human health. Herein, we present a longitudinal paired study across the swine lifetime from birth to market, coupled with metagenomic sequencing to explore the dynamics of ARGs and their health risk in the swine fecal microbiome.

**Results:**

We systematically characterized the composition and distribution of ARGs among the different growth stages. In total, 829 ARG subtypes belonging to 21 different ARG types were detected, in which tetracycline, aminoglycoside, and MLS were the most abundant types. Indeed, 134 core ARG subtypes were shared in all stages and displayed a growth stage-associated pattern. Furthermore, the correlation between ARGs, gut microbiota and mobile genetic elements (MGEs) revealed *Escherichia coli* represented the main carrier of ARGs. We also found that in most cases, the dominant ARGs could be transmitted to progeny piglets, suggesting the potential ARGs generation transmission. Finally, the evaluation of the antibiotic resistance threats provides us some early warning of those high health risk ARGs.

**Conclusions:**

Collectively, this relatively more comprehensive study provides a primary overview of ARG profile in swine microbiome across the lifetime and highlights the health risk and the intergenerational spread of ARGs in pig farm.

**Graphical Abstract:**

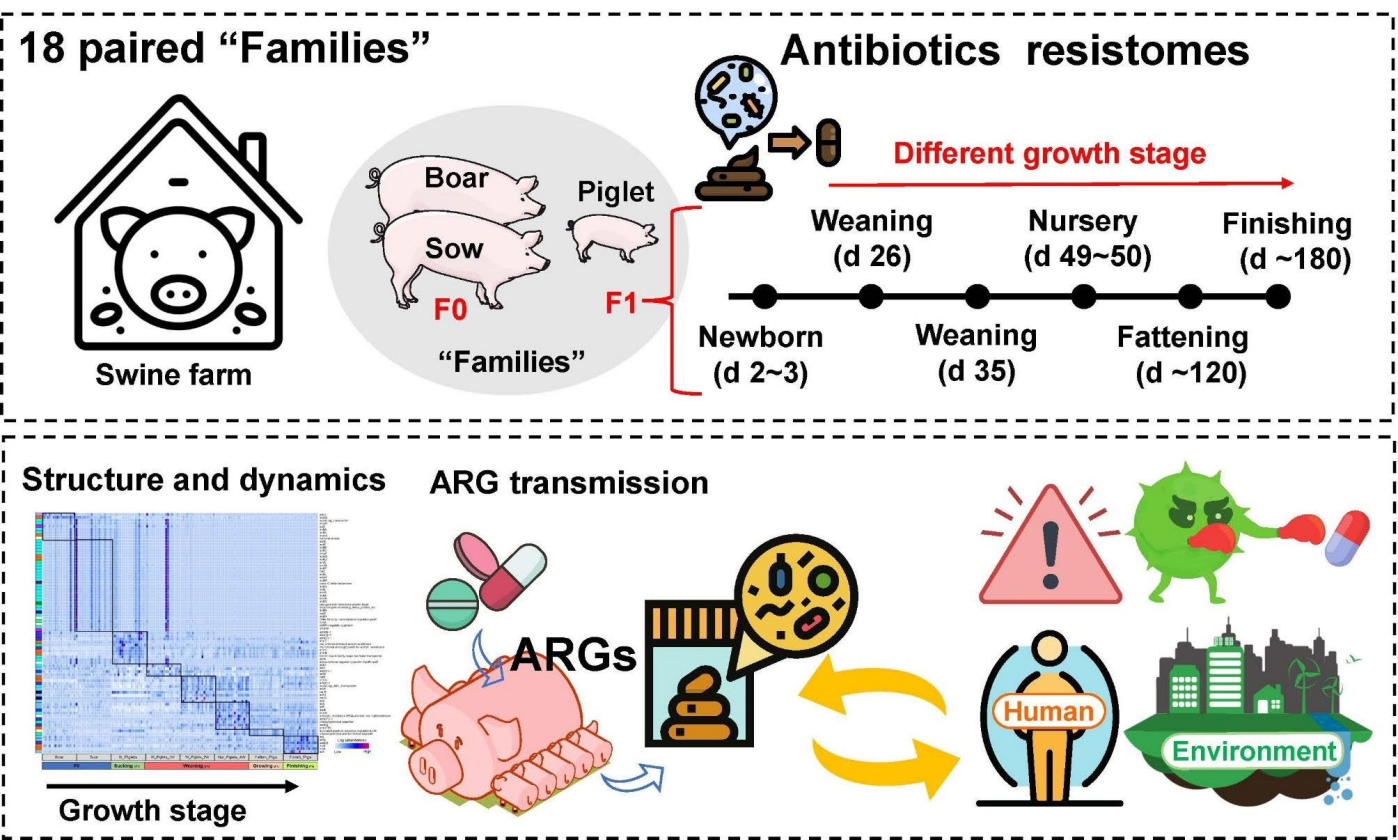

**Supplementary Information:**

The online version contains supplementary material available at 10.1186/s42523-023-00279-z.

## **Introduction**

Globally, an estimated 73% of antibiotics are used in food-animal production [1]. Worldwide antimicrobials use facilitates the occurrence of antimicrobial resistance (AMR), posing a substantial threat to animal and human health [2–4]. It is predicted that AMR now causes 700,000 or more deaths every year and could further grow to 10 million by 2050 [5, 6]. The occurrence of AMR is largely driven by antibiotics selection pressure and promotes the mobilization and horizontal transfer of antibiotic resistance genes (ARGs), the emerging environmental contaminants [7, 8]. The concurrent spread of ARGs by mobile genetic elements (MGEs) could occur between human, animal and the environment, which further aggravated the persistence and spread of ARGs [9].

Gut microbiota is considered a reservoir of ARGs [10, 11]. Studies have suggested the close associations between the bacteria and human or animal gut antibiotics resistome [12–14]. For example, *Escherichia coli* (*E. coli*), as a widespread commensal in the gastrointestinal tract of both humans and animals, is widely studied as a key indicator bacterium carrying drug-resistant genes [8, 15]. Long-term use of antibiotics in food animals even at low doses could accelerate the production of antibiotic-resistant bacteria (ARB) [16]. In turn, the antibiotic resistance of ARB from animals can be transferred to humans, causing a range of health impacts [6, 17].

Swine farms are considered as the potential hot spot for the dissemination and development of ARGs. Pigs as an important source of ARGs, accounting for 52.2% of the total antimicrobial usage in China [18]. The characterization of the swine gut resistome at various gut locations or farm locations has provided references for optimizing the use of antimicrobials in pigs [19, 20]. Recently, human studies indicated that ARG composition is altered at different ages, highlighting that age needs to be considered a critical factor for ARGs studies [21]. Furthermore, human maternal gut microbes harbour ARGs able to transfer to newborn infants during or shortly after birth, which reveals the mother-to-child ARG transmission pattern [22, 23]. Although recent technological advances have promoted a rapidly increasing number of swine gut resistome research, systematic longitudinal research on the transmission of the swine gut resistome from both maternal and infant generations or the dynamics of ARG profiles across the different growth ages is lacking.

Here, we present a paired longitudinal study of 144 fecal samples from F0-F1 generation across the swine lifetime from birth to market, coupled with metagenomic sequencing to bridge this knowledge gap. We systematically characterized the composition and distribution of ARGs among the different growth stages in the swine microbiome and identified a different growth stage-associated ARGs pattern. Furthermore, we determined the link between ARGs and gut microbiota, identifying that *E. coli* might as the main host microbe of ARGs. We further found that in most cases, the dominant ARGs of the F0 sow generation might be transmitted to infant piglets, which might be associated with gut bacterial transmission. Finally, the evaluation of the antibiotic resistance threats during each growth stage of pig farms provides us with some early warning of those related high-risk ARGs.

## Results

### Distribution and abundance of ARGs among the different growth stages in pigs

**Fig. 1:**
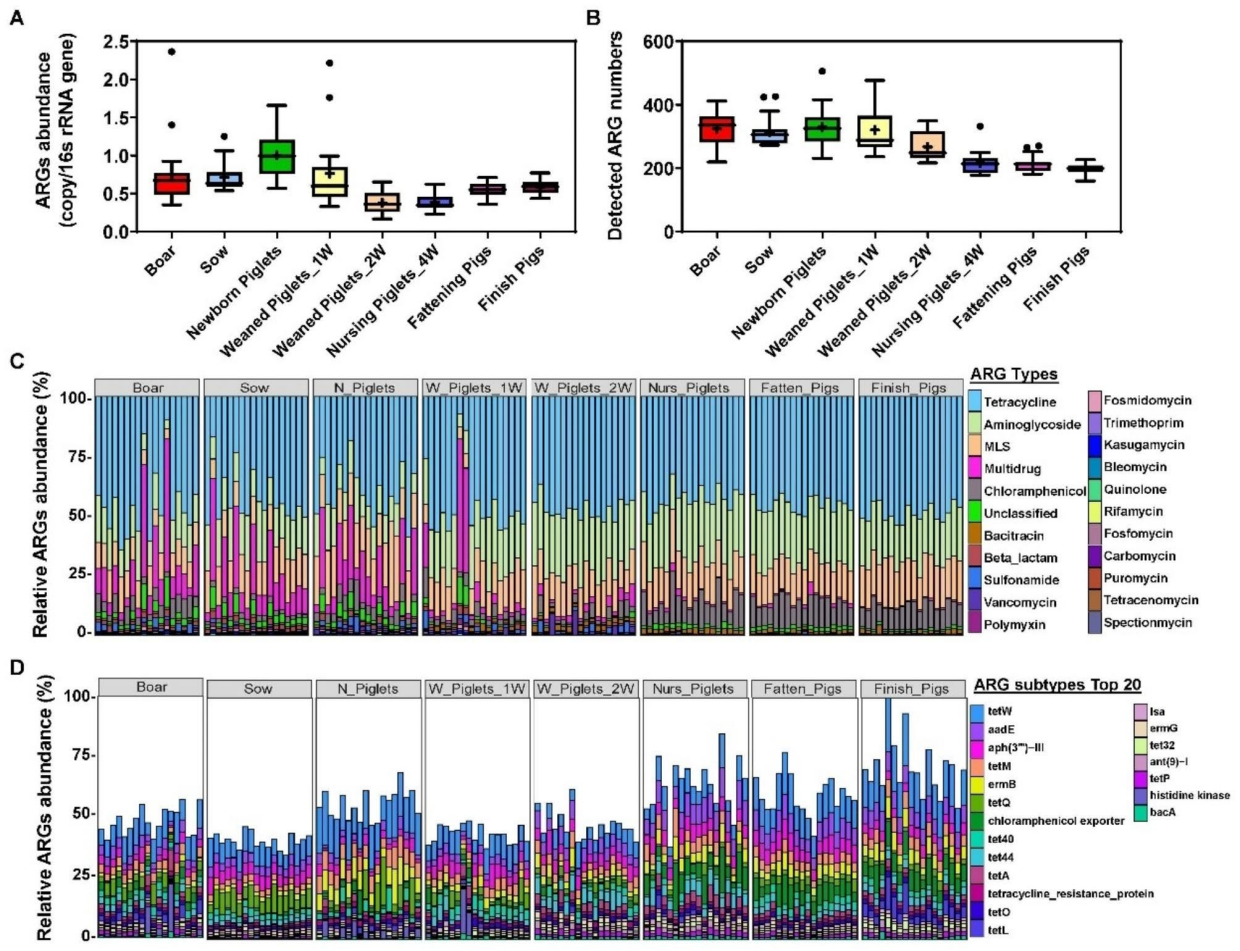
Characteristics of ARGs among the different growth stage in pigs. **(A)** Box plot showing the total ARGs type abundance (copy/16s rRNA gene) in each growth stages. **(B)** Detected ARG subtypes numbers in each growth stages. **(C)** Distribution of relative ARG abundance in pigs with different types. **(D)** Top 20 ARGs subtypes based on the abundance in each sample

PCoA based on the relative abundance of ARGs revealed a significantly different ARG pattern between pig samples (Fig. S2). For example, the ARG patterns in newborn piglets were found to be more discrete than those in other stages, which might have resulted from environmental exposure (Fig. S2). Additionally, weaned pigs showed a tendency to the finishing stage (Fig. S2). The ARG abundance (copies per 16 S rRNA gene) was significantly higher in pigs of the F0 generation and F1 newborn piglets (Fig. 1A). Furthermore, the abundance of ARGs was obviously decreased at the post-weaning stage but slightly increased from the nursing stage to the finishing growth stage in pig samples (Fig. 1A, Table S1). In contrast, the changes in the detected ARG subtype number decreased from the weaning to the finishing growth stage (Fig. 1B, Table S1).

In general, these ARGs were divided into 21 main ARG types, in which aminoglycoside, tetracycline, and MLS were the most dominant types specifically to “W_Piglets_1W to Finish_Pigs stages, accounting for approximately 80% of the total abundance; additionally, the predominant ARG types for the Boar, Sow, and N_Piglets were aminoglycoside, tetracycline, and Multidrug (Fig. 1C). Moreover, the F1 generation of newborn piglets showed ARG type patterns similar to those of the F0 generation of boar and sow samples (Fig. 1C). From the nursing stage to the finishing growth stage, the abundance of chloramphenicol increased (Fig. 1C). However, the richness of the AMR class was decreased in those stages, consistent with the reduced ARG numbers (Fig. 1C). In addition, the multidrug level was much higher in the boar, sow and newborn piglet groups (Fig. 1C). The general ARG type pattern remained similar during the fattening and finishing stages (Fig. 1C).

A total of 829 ARG subtypes conferring resistance to 21 different ARG types were further detected. Subtypes with abundances greater than 0.1% were identified as dominant ARGs, which included 98 subtypes and accounted for 93.8% of the total abundance. The top 20 most abundant dominant ARG subtypes are displayed in stacked bar charts in Fig. 1D. Among these ARG subtypes, 11 belong to tetracycline resistance genes, including *tet*W, *tet*M, *tet*Q, *tet*(40*)*, *tet*(44), *tet*A, *tet*O and *tet*L, the most dominant ARG class throughout most of the stages, especially in the nursery, growing and finishing stages (Fig. 1D). Furthermore, 3 aminoglycosides resistance subtypes (*aad*E, *aph*(3)*-*III, *ant*(9)*-*I) were also enriched in almost all growth stages (Fig. 1D).

### Different resistance mechanisms among the different growth stages in pigs

We further identified resistance mechanisms for ARG subtypes, which cover 4 different resistance mechanisms (Fig. S3). As shown in Fig. S3, the two predominant mechanisms among the samples were resistance to antibiotic inactivation and antibiotic target protection. In particular, the ARGs that confer resistance to multiple antibiotics using efflux mechanisms were more abundant in the F0 boar, newborn piglets, and weaned piglets than in other stages (Fig. S3).

### Core and stage-associated ARGs among the different growth stages in pigs

ARG subtypes presenting in at least 95% of the samples were defined as core ARGs, and a total of 134 core ARG subtypes belonging to 17 ARG types were shared in all the samples. The co-occurrence patterns of the ARG subtypes were explored using network inference with strong (Spearman’s correlation coefficient (ρ) > 0.9) and significant (*p*-value < 0.01) correlations (Fig. S4). Fig. S4 consists of 51 nodes (ARG subtypes) and 455 edges. Based on the modularity class, the entire network could be parsed into five major modules. The multidrug resistance genes formed the largest module I, whereas ARGs resistance to aminoglycoside formed the module II, in which *Aph* (6’)-I was the hub; In addition, tetracycline resistance genes, including *tet* (X) formed Module III (Fig. S4).

**Fig. 2:**
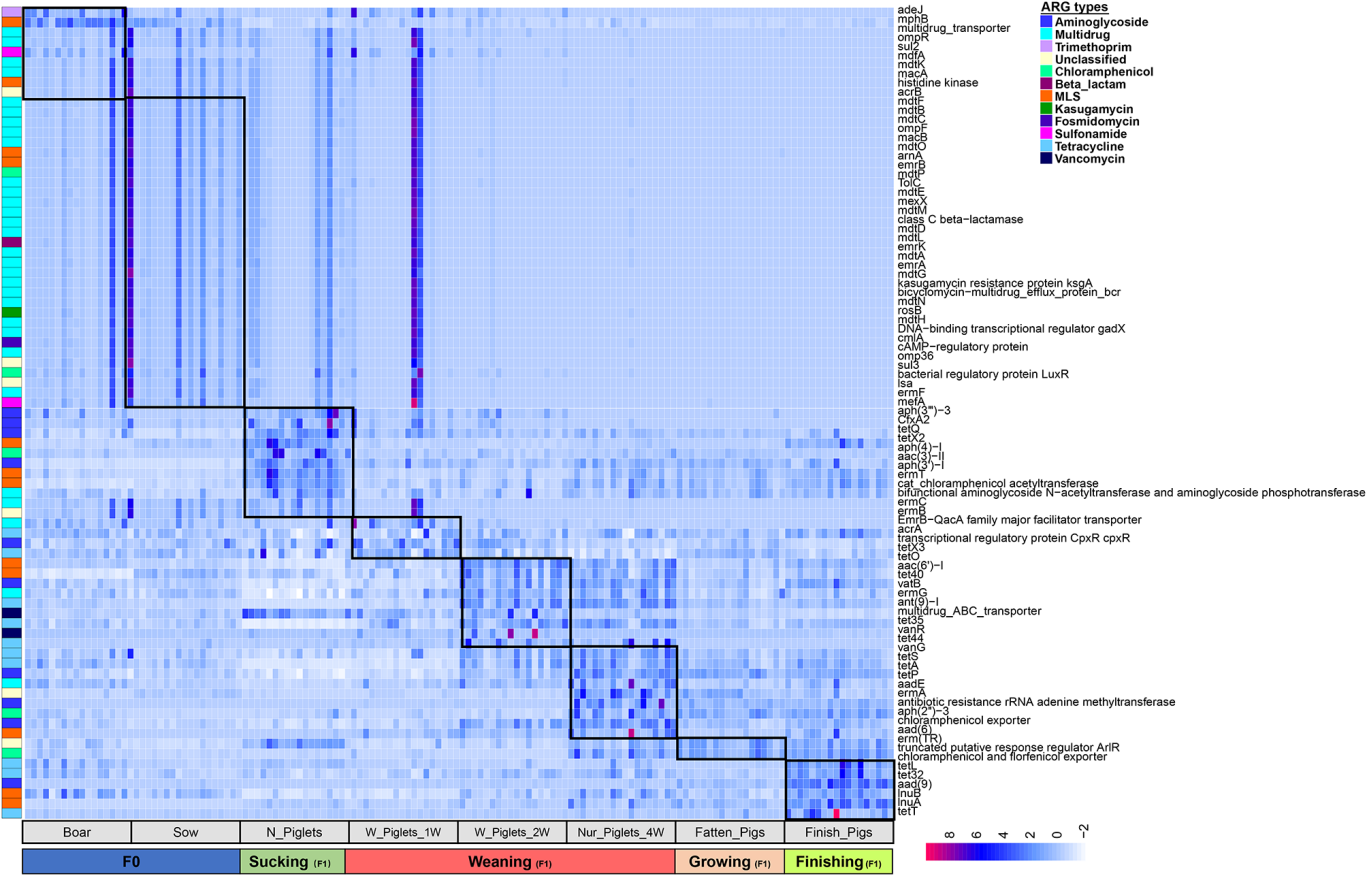
The stage associated ARGs among the different growth stage in pigs. Heat map shows the 90 stage-associated ARG subtypes identified by LEfSe (LDA > 3) among samples. Heat map shows the normalized average abundances based on the row. The left line means the ARG types in which those ARGs subtypes belonging to

Stage-associated ARGs were further identified by using LEfSe as visualized on a heatmap (Fig. 2). As shown in Fig. 2, although the core ARG subtypes persisted throughout all stages, their presence also followed a stage-associated pattern. For instance, the trimethoprim resistance gene *ade*J, multidrug resistance genes *mph*B, *mdf*A and *mdt*A, and MLS resistance genes *mph*B and *mar*B were abundant in the F0 generation of boar and sow samples but remarkably lower after weaning at subsequent stages. The aminoglycoside resistance genes were higher in the weaning stage for 1 to 4 weeks but decreased in subsequent stages. The abundance of tetracycline resistance genes increased from the nursing stage to the finishing stage.

### Microbiome profiles among the different growth stages in pigs

**Fig. 3:**
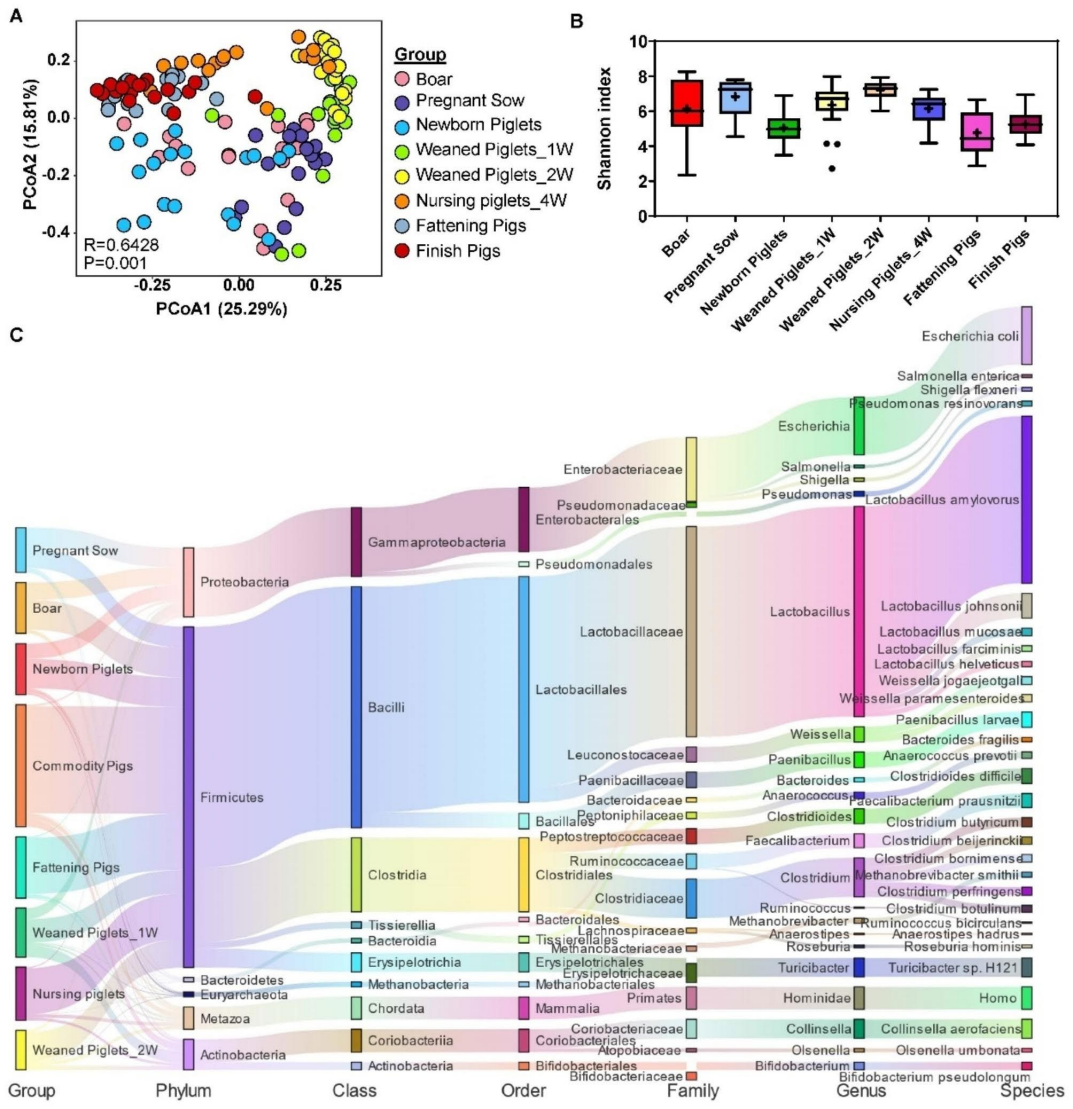
Microbiome profiles among the different growth stage in pigs. **(A)** PCoA plots show the distinct clusters in each group. **(B)** Boxplot showing the microbial richness of Shannon index in each group. **(C)** The distribution of bacteria at different taxonomy tax from phylum to species levels. The colors of the rectangles represent different bacteria

Significant shifts in the bacterial community and structure among experimental pigs from all stages were observed in the PCoA plots based on Bray-Curtis distance (Fig. 3A). For the different growth stages, fattening and finishing stage pigs showed a distinct different bacterial community pattern (R value = 0.3669; *P* value = 0.001), and the microbiota profiles in nursing piglet samples was also significant different with the fattening and finishing stage samples (R value = 0.4161; *P* value = 0.001). Additionally, the newborn samples were distinct from those and more similar to the F0 sow samples (R value = 0.6772; *P* value = 0.001). The overall alpha richness significantly increased over time starting from the birth stage and decreased from the nursery stage, as demonstrated by the Shannon index (Fig. 3B, Table S1). However, the alpha diversity of the Chao 1 index increased in the postweaning stages (Fig. S5A-B). Newborn piglets that harboured fewer gut microbes showed lower bacterial diversity and richness (Fig. 3B and Fig. S5A-B).

At the phylum level, Firmicutes was the dominant phylum in all samples, which increased from the weaning to the finishing stages (Fig. 3C and Fig. S5C). The relative abundance of Proteobacteria decreased during the newborn to finishing stages (Fig. S5C). In addition, Actinobacteria levels were higher in the piglet stage, especially in the weaned pig samples. Moreover, F0 generation samples showed higher levels of Bacteroidetes. At the genus level (Fig. S5D), *Lactobacillus*, *Escherichia*, and *Clostridium* were the top three genera in all samples. *Lactobacillus* was more abundant in the fattening and finishing stage samples. Pathogens such as *Escherichia* were higher in the F0 generation, newborn and finishing stage samples (Fig. S5D).

Next, correlation analyses between microbiota communities and ARGs were conducted to identify key bacteria that affecting the diversity of ARGs. In this study, the most abundant phyla Firmicutes was found to be significantly correlated with almost all the types of ARGs and cooccurred with sulfonamide, beta-lactam, quinoline, trimethoprim, multidrug, polymyxin, fosmidomycin and kasugamycin resistance genes (Fig. S5E). In addition, Firmicutes was positively associated with aminoglycosides, bacteria, chloramphenicol, tetracycline, bleomycin, and MLS, which was similar to a previous report [24]. In contrast, Proteobacteria and Bacteroidetes were positively linked to ARGs, such as beta-lactam, quinoline, trimethoprim, and multidrug, indicating that Proteobacteria and Bacteroidetes might harbour these ARGs (Fig. S5E).

### Distribution and abundance of MGEs among the different growth stages in pigs

The distribution and total abundance of MGEs among experimental pigs are shown in Fig. S6. The boar and sow F0 generation contained higher MGE abundances than the other groups. Furthermore, the detected MGE numbers decreased in the postweaning stages (Fig. S6B). As shown in the stacked bar charts, the dominant MGEs in all samples belonged to transposase and IS (Fig. S6C). TnpA, a transposase, was the most dominant MGE subtype among all the samples, especially in the finishing stage (Fig. S6D). In addition, weaning stress might increase the abundance of IS10 and tnpAB, as they were higher in the weaned piglets (Fig. S6D).

### Co-occurrence patterns between ARGs, MGEs and bacteria

**Fig. 4:**
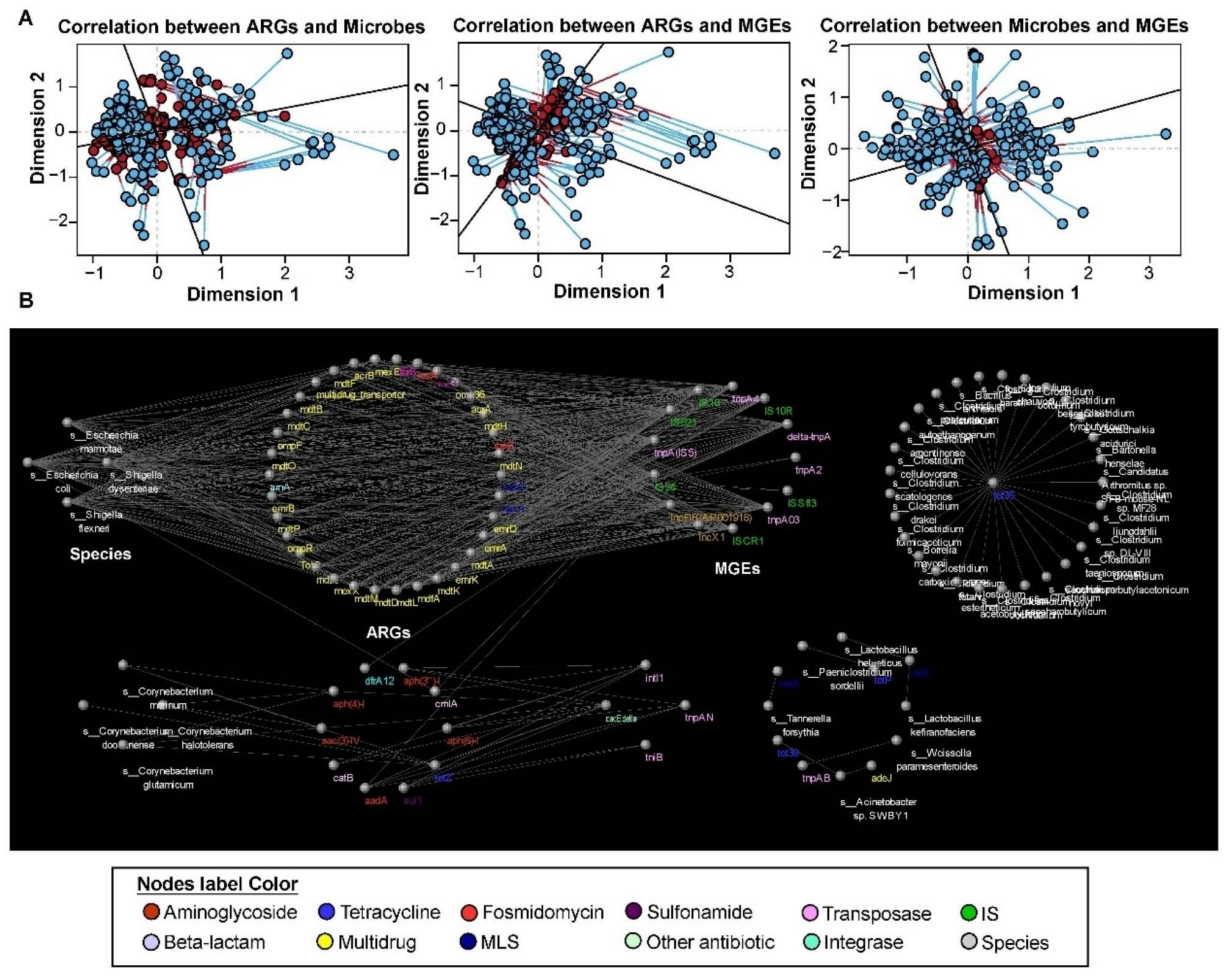
Co-occurrence patterns between ARGs, MGEs and bacteria species. **(A)** The association in the abundance of ARGs and microbes, ARGs and MGEs, microbes and MGEs by Procrustes analysis and Mantel test. **(B)** Network analysis, the nodes were coloured according to different ARGs subtype, MGEs subtypes and bacteria species with the correlation of a strong (Spearman’s correlation coefficient (ρ) > 0.9) and significant (*p*-value < 0.01) correlation. Node size representing the number of connections and edges representing the correlation coefficient

To further explore the association between the microbial community, ARGs and MGEs, we used Procrustes analysis to correlate these profiles (Fig. 4A and Table S2). The results showed that ARG profiles were significantly related with the bacterial community (Mantel_r = 0.774, Proc_r = 0.597, *p* < 0.001) and MGEs (Mantel_r = 0.520, Proc_r = 0.620, *p* < 0.001). Additionally, MGEs were significantly associated with the bacterial community (Mantel_r = 0.374, Proc_r = 0.809, *p* < 0.001) (Fig. 4A and Table S2).

The co-occurrence network among core ARGs, MGEs and bacterial species consisted of 116 nodes and 526 edges (Spearman correlation > (0.9), *p*-value < 0.01). In this regard, pathogens belonging to the *Shigella* and *Escherichia* genera (*Shigella dysenteriae*, *Shigella flesneri*, *Escherichia marmotae* and *E.coli*), multidrug resistance genes (such as *mdtB*, *mdtE*, *mdtF*, *mdtM*, *ompR*, and *ompF*), MLS resistance genes (such as *macA*, *mexE* and *mexX*) and MGEs (such as IS10, IS62, IS26 and IS CR1) formed the largest module, suggesting interactions among them (Fig. 4B). *S.dysenteriae*, *S.flesneri* and *E.coli* may be carriers of IS62, IS26 and ISCR1. In some other modules, most bacterial species displayed a stronger association with only one specific ARG subtype. For example, *Clostridium* spp. displayed a significant correlation with *tet(35)*, which is a tetracycline resistance gene. In addition, *Tannerella forsythia*, *Paeniclostridium sordellii* and *Acinetobacter* sp. SWBY1 may act as a potential host of *mef*A, *tet*P, *tet*39 and *ade*J. The results also showed that *Weissella paramesenteroides* might act as the carrier of tnpAB.

### *E.coli* associated with ARGs distribution in pigs

**Fig. 5:**
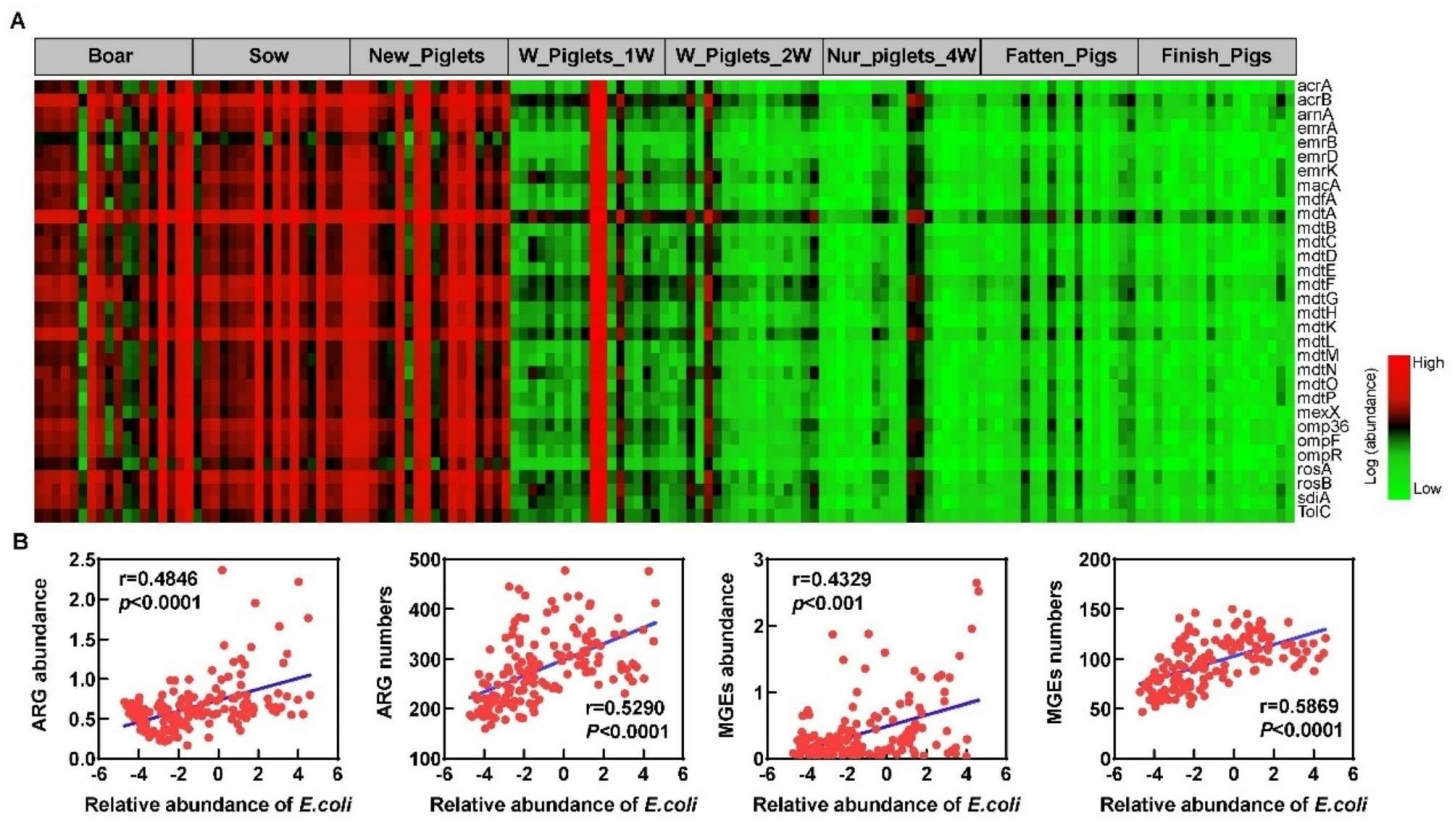
*E.coli* associated with ARGs distribution in pigs. **(A)** Heatmap showing the abundance of these ARGs in pigs among different growth stages. **(B)** The correlation between the abundance of *E. coli* with the ARG and MGEs abundance, number

Since we found *E. coli* and *Shigella* spp. strongly associated with most ARGs in the pig samples, we identified those *E. coli*-associated ARGs as potential risk ARGs. The heatmap showed that potential risk ARGs were mainly enriched in the F0 generation and newborn samples. In Fig. 5A, we found the relative abundance of *E. coli* to be associated with most of the variation of the ARGs (Fig. 5A). We further plotted the relative abundance of *E. coli* (log10) versus the abundance and number of ARGs and MGEs from all samples to directly determine the association between *E. coli* and overall resistome outcomes. The scatterplots show a strong association between *E. coli* and ARG and MGE abundance and detected numbers across different growth stages (Fig. 5B).

### Health risks of ARG evaluation among the different growth stages in pigs

**Fig. 6:**
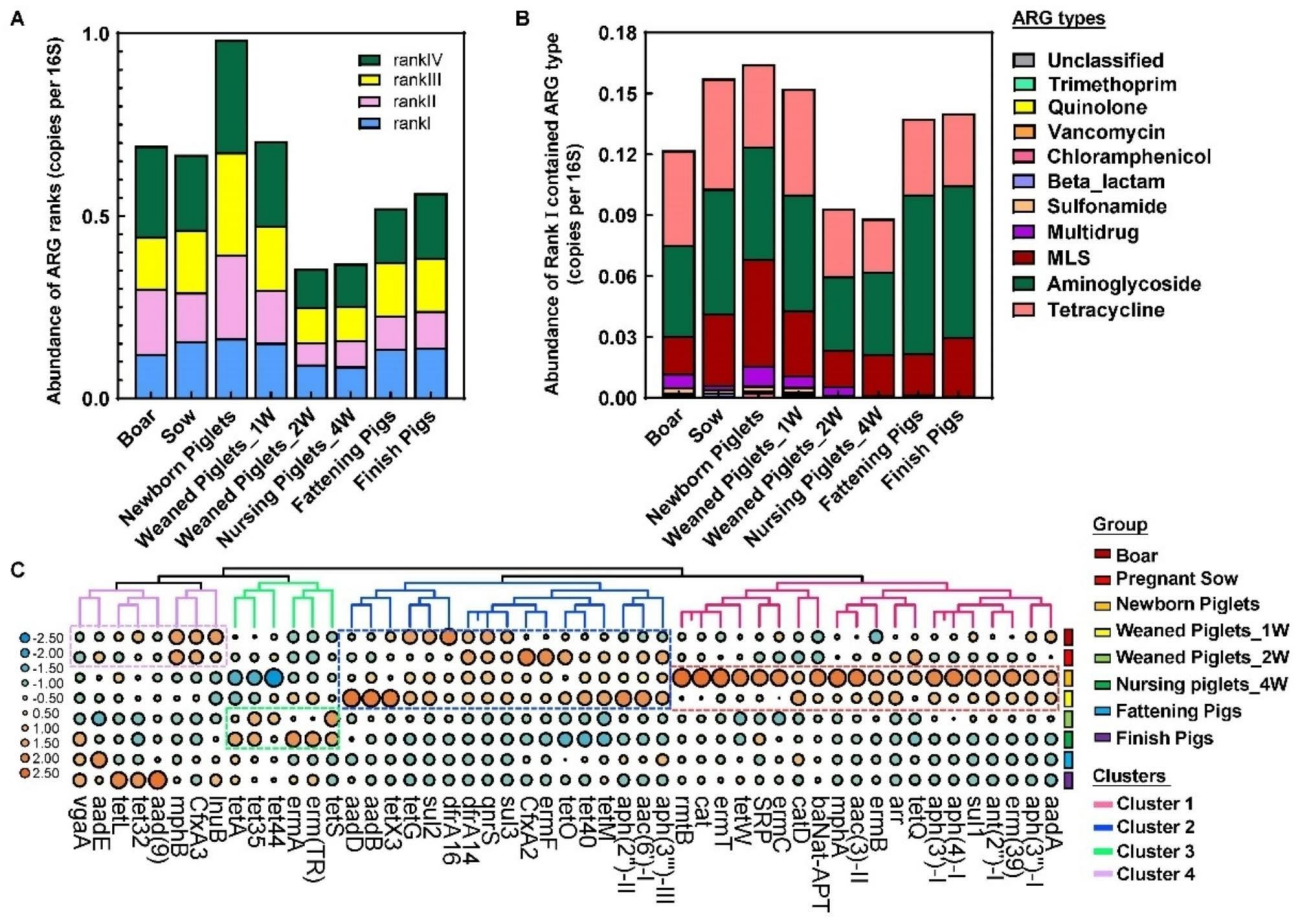
ARGs health risks evaluation among the different growth stage in pigs. **(A)** The distribution of Rank risk ARGs among each group. **(B)** Abundance of Rank I risk ARGs among each group. **(C)** Top 50 of identified Rank I risk ARGs subtypes based on abundance. Different color cluster means the different Rank I risk ARG subtypes pattern among growth stages

Next, ARG risk rank analysis was performed to evaluate ARG risk and to identify the ARGs that have significant potential to endanger public health [25]. As shown in Fig. 6, the highest risk Rank I (already present in pathogens of ARGs)-associated ARGs were increased during the early life of newborn piglets but decreased in the nursing stage. However, it increased during the fattening and finishing stage. In particular, high-risk ARGs were highest in newborn piglets. In addition, rank I contained tetracycline, aminoglycoside, and MLS resistance ARGs. The top 50 high-risk ARGs in all growth stages are shown in Fig. 6C. We found that Cluster 2, including tetracycline and aminoglycoside resistance genes, especially *aph*(3)-III, *tet*M, *tet*O, *tet*40, *sul*3, *qnr*S and *dfr*A 14, displayed similar patterns between the F0 and F1 early-life pigs, indicating that those ARGs might be transmitted from the sow mother generations. However, we also found that *rmt*B, *erm*T, *tet*W, *erm*C, *mph*A, *aph*(3), *sul*1, and *erm*(39) were enriched in the newborn piglets but were missing in the F0 generation (Cluster 1) (Fig. 6C). During the growth and finishing stages, the health risk slightly increased, which might have resulted from the increased abundance of *aad*(9), *tet*32, *tet*L, *aad*E and *vga*A (Cluster 4) (Fig. 6C).

## Discussion

In the present study, a relatively more comprehensive metagenomic analysis was conducted to explore ARG profiles of the swine microbiome across different growth stages, including the F0 generation of boars, sows, newborn piglets, weaning piglets, nursery piglets, fattening pigs and finishing pigs. We systematically characterized the composition and distribution of ARGs among the different growth stages in the swine microbiome. In total, 829 ARG subtypes belonging to 21 different ARG types were detected. Aminoglycoside, tetracycline, and MLS were the most abundant ARG types in pig samples. A total of 134 core ARG subtypes belonging to 17 types were shared in all samples and displayed different growth stage associated ARG patterns. Furthermore, the link between ARGs, gut microbiota and MGEs suggested an association between ARGs and gut microbiota, in which *E. coli* might as the main host microbe. Interestingly, we found that in most cases, the dominant ARGs of the F0 sow generation of sows’ dominant ARGs might be transmitted to infant piglets, which might be associated with gut bacterial transmission. Finally, the evaluation of the antibiotic resistance threats during each growth stage of pig farms provides us with some early warning of high-risk ARGs.

### The dynamics of the antibiotic resistomes in swine gut microbiome

Previous studies have revealed that the pig fecal resistome varies among farms, which might result from different locations, housing conditions, weather climates and environments [3, 26]. The ARG types mainly conferred resistance to tetracycline, aminoglycosides, and MLS. In our study, we found that the majority of ARG types of fecal resistomes from all growth stages also belonged to tetracycline, aminoglycoside, MLS and multidrug. A study focused on the fecal resistome of dairy cattle suggested that dynamic changes in the resistome are closely associated with diet transition [27]. In addition, human study identified the age-specific associated antimicrobials within children, adults and elderly individuals, highlighting the association between age groups and antimicrobials used during each age stage [28]. Thus, the ARG profiles in abundance and richness differed between different growth stages in swine microbiome, might be caused by diet, antimicrobial use and ages in this study.

After the weaning stage, pigs appeared more resistant to tetracycline and aminoglycosides. Tetracyclines were used the most to treat the respiratory organs of fattening and postweaning pigs, which explained why we found that the abundance of tetracyclines was increased from the weaning stage in our study [29]. In addition, fecal ARGs of the nursing, fattening and finishing stages showed resistance to chloramphenicol, which is also commonly used for disease prevention in pig farms. Despite the decrease of abundance in total ARGs, an overall increasing ARG subtypes number was observed during the lifetime of pigs, raising concerns that ARG richness was increased with age.

From the stage associated ARG subtypes, we found that resistance genes conferring multidrug resistance were more abundant in the F0 generation sow samples, which may be due to the long-term use of antibiotics in sows, particularly during farrowing. Oral medication in suckling and post-weaning periods were the most common applications of antibiotic administration in pig production. Considering the phase of pig, the most used was colistin in piglets and weaners, and tylosin in fatteners for gastrointestinal conditions [30, 31]. Pleuromutilin were commonly used for respiratory tract infections in sow and piglets [32]. Although the different ARG subtypes were enriched in distinct growth stages, the co-occurrence pattern revealed that the hubs and related cooccurring ARGs in each module might be harboured in some specific bacteria taxa that might be shared within different growth stages.

### The gut microbiota composition contributed to the occurrence of ARGs

Increased gut microbial diversity (Shannon index) from newborn to 2 weeks postweaning was found in this study, consistent with previous studies [27]. We found that pig guts harboured a lower diversity of microbiota and fecal resistomes but higher ARG abundance, consistent with the guts of patients who underwent antibiotic treatment [33]. However, richness indices (number of observed species) continued to increase until the end of the experiment. These results suggest that antibiotic administration could reduce the diversity of the bacteria, promote the enrichment of ARB, further accelerating the rise of AMR levels.

There are more concerns about some human-associated pathogens, especially those potentially carrying antibiotic resistance. The bacterial species that showed a strong association with ARG subtypes may be regarded as potential ARG carriers [34]. For example, *E. coli* are pathogens inhabiting the intestinal tract of humans and animals that have been reported to harbour the largest number of ARGs, showing multidrug, fluoroquinolone and macrolide resistance [35, 36]. We also found that the *E. coli*-associated ARGs were resistant to tetracycline and other antibiotics. *Shigella* is another major pathogen that causes diarrheal diseases in humans and animals [37]. Our data showed that *Shigella* species (*S*. *dysenteriae* and *S*. *flexneri*) were related to the majority of ARGs similar to *E. coli*, indicating the health risk of those-associated ARGs to humans and animals.

We also found some ARGs were carried by multiple bacteria. The gene *tet* (32) was identified in the Clostridium-related bacterium. Herein, *tet*(35) was found to be associated with *Clostridium* spp., suggesting that the *Clostridium*-like strain might also be the carrier of *tet* (35). Aminoglycoside resistance genes such as *aph* and *aad* were determined to be related to *Corynebacterium*-related species. Aminoglycosides are second-line or complementary antibiotics used for the treatment of pathogenic *Corynebacterium* infections [38]. We thus proposed that the long-term use of aminoglycosides enhanced AMR risks. On the other hand, *Lactobacillus*-related species, such as *L. helveticus* and *L. kefiranofaciens*, with potential benefits were also identified as ARGs carriers, suggesting that the use of potential probiotics in pig production also needs to be done with caution.

### The potential ARGs transmission patterns from the F0 to F1 might associate with bacterial transmission

Another important finding in our study is that we found the potential “mother-to child” ARGs transmission in pigs. Human studies previously reported mother-to-child bacterial transmission events [23]. Evidence also proved that maternal gut microbes harbouring ARGs are transferred to newborn infants during the first few days after birth [22, 23]. In the present study, *E. coli*-associated ARGs in newborn piglets were similar to those in sow samples. *E. coli*, which was highly abundant in the sow sample, was abundant in newborns but phased out after weaning in our study. These results suggest that the F0 generation of sows’ dominant ARGs might transmit to infant piglets, which might be associated with gut bacterial transmission patterns such as *E. coli*. However, the direct isolation of *E. coli* and the identification of ARGs carried still need further confirmation to support our hypothesis.

Some co-occur high risk ARG, such as *aph*(3)-III, *aac*(6)-I, *aph*(2)-II, and *tet* (M/O/G) were found in the F0 generation and newborn piglets but rarely in other stages, also suggesting their vertical inheritance. The examination of “mother-to-child” ARGs transmission brings us closer to understanding the maternal transmission and their contributions to the infant ARGs profile changes, remaindering us of to focus on maternal antibiotic use. However, larger, well-balanced cohorts still needed to confirm the ARGs or microbial acquisition patterns in further studies.

### The evaluation of the antibiotic resistance threats during each growth stage

Current research on pig resistance genes is mainly based on composition or abundance; however, the health risk of ARGs should also be taken into consideration [25, 39, 40]. Study had developed an omics-based framework to evaluate the health risk of ARG by considering human-associated enrichment, gene mobility, and host pathogenicity [25]. Our results demonstrated that 21.3% of the ARGs pose the highest health risk, especially those conferring beta-lactam and multidrug resistance. Among these, high-risk ARGs, such as *aph*(3)-I, *aac*(3)-II, *tet*Q, *tet*L, *aph*(6)-I, and *aph*(2)- II, were also identified by the World Health Organization (WHO), warning that these ARGs may be “current threats”, which have the highest potential to result in multidrug resistance in pathogens. The F0 generation of boars and sows was often used for breeding and has been raised for 2 years. It is reasonable that we found that the F0 generation of boars and sows showed the highest health risk due to the long-term use of antibiotics. Previous study revealed that animal exposure may as a potential determinant for resistome or microbiome composition in interconnected humans [14]. The evaluation of the antibiotic resistance threats during each growth stage in pig farms provides us with some early warning of those high-risk ARGs. In other words, these high-health-risk ARGs also provide potential markers for evaluating the risk by simplified methods (e.g., quantitative PCR).

In conclusion, the pig ARG profiles exhibited a growth-stage associated patterns, which partly due to the diets and gut microbiota changes. Some ARGs co-occur in F0 generation and newborn piglets, suggesting their inheritance. Apparently, more attention should be paid to the health risk of ARGs in pig farms since these ARGs could be easily transferred to zoonotic pathogens. Collectively, this relatively more comprehensive study provides a primary overview of ARG profiles in swine microbiome across the lifetime and highlights that the risk of ARGs and intergenerational impact of antibiotics should not be ignored.

## Materials and methods

### Study design and sample collection

The pig farm is located in Lin’an District, Hangzhou city, Zhejiang province, China (30°14′10″N 119°42′55″E). In this study, 18 boars and 18 sows were used as F0 generation. And total of 18 samples (one piglet from each F0 family based on the average bodyweight) were identified as F1 (Fig.S1). Among these pigs, 18 male pigs were paired across the different growth stages, including newborn (d 2 ~ 3), two weaning phases (d 26 for weaning 1 week and d 35 for weaning 2 weeks), nursery (d 49 ~ 50), fattening (d ~ 120) and finishing stage (d ~ 180). Rectal swabs were collected at the end of each phase. Related sample information has been listed in Table S3.

### Metagenomic sequencing

Microbial DNA of the total 144 samples was extracted according to the manufacturer’s instructions using a E.Z.N.A.® DNA Kit (Omega Bio-tek, Norcross, GA, USA). Metagenomic sequencing libraries were performed at the Shanghai Biozeron Biological Technology Co. Ltd. (Shanghai, China). All samples were sequenced on Illumina Novaseq 6000 (Illumina, Inc.San Diego, California USA). Adaptor contaminants and low-quality reads were removed by Trimmomatic (V 0.32) [41]. Bwa2 was used to remove host DNA. And the reference genome was Sus scrofa genome assembly Sscrofa11.1. Reads with host genome contamination and low-quality data removed are called clean data and used for further analysis.

### Bacterial taxa, ARGs and MGEs annotation

For the quantification of ARGs, the reads were used as inputs to the ARG-analysis pipeline ARGs-OAP v2.0, integrating detection of ARGs by the SARG v2.0 reference database [42, 43]. ARGs was normalized by the number of 16 S rRNA genes, which were expressed as copies per 16 S rRNA gene. The related information of ARG types and subtypes in the present study have shown in Table S4-S5. For the quantification of MGEs, the reference database with the published MGEs database was used [44]. It contains 278 different genes and more than 2000 reference sequences. We classify all genes based on their gene structures and evolutionary relationships. Mobile gene units are divided into two levels: type and subtype, where type is the type of mobile gene unit (such as plasmid, integron, etc.), and subtype is a specific gene (such as intI1, tnpA, etc.). The MGEs was also normalized by the number of 16 S rRNA genes. The related information of MGE type and subtypes in the present study have shown in Table S6-S7. For taxonomic classification, reads were cleaned by Kraken2 (v2.0.7) with NCBI taxonomical ID, and a customized complete genome k-mer database [45]. The classification results were further passed through with Bracken 2.0 [46].

### ARGs health risk evaluation

The health risk assessment of ARGs was evaluated according to the criteria proposed by [25] according to these perspectives: (1) enrichment in human-associated environments; (2) gene mobility; (3) presence in host pathogenicity. ARGs were classified into four ranks ARGs: (1) Rank IV (lowest risk): ARGs that do not meet the first criterion; (2) Rank III: ARGs that meet the first, but not the second; (3) Rank II: ARGs that meet the first and second but not the third; (4) Rank I (the highest risk): ARGs that meet all three criteria.

### Statistical analysis

Statistical analysis was performed by R (Version 4.0.2). Principal coordinates analysis (PCoA) based on Bray-Curtis was used to determine beta-diversity of bacterial communities and ARGs distribution among groups. Procrustes analysis was used to assess the congruency of sample separations within the bacteria species, ARG and MEG profiles. The Spearman correlation coefficient of ARG types was performed to analyze the correlation. Network visualization was performed using Cytoscape 3.8.2 and Gephi 0.9.2 [47].

### Electronic supplementary material

Below is the link to the electronic supplementary material.

Supplementary Material 1

Supplementary Material 2

Supplementary Material 3

Supplementary Material 4

Supplementary Material 5

Supplementary Material 6

Supplementary Material 7

## Data Availability

Metagenomic data are available on the NGDC (https://ngdc.cncb.ac.cn/, GSA No.CRA010380).

## References

[1] Van Boeckel TP, Glennon EE, Chen D, Gilbert M, Robinson TP, Grenfell BT, Levin SA, Bonhoeffer S, Laxminarayan R (2017). Reducing antimicrobial use in food animals. Science.

[2] Tiseo K, Huber L, Gilbert M, Robinson TP, Van Boeckel TP. 2020. Global trends in Antimicrobial Use in Food animals from 2017 to 2030. Antibiot (Basel) 9. 10.3390/antibiotics9120918 PMC7766021 33348801

[3] Zhou Y, Fu H, Yang H, Wu J, Chen Z, Jiang H, Liu M, Liu Q, Huang L, Gao J, Chen C (2022). Extensive metagenomic analysis of the porcine gut resistome to identify indicators reflecting antimicrobial resistance. Microbiome.

[4] Okoye CO, Nyaruaba R, Ita RE, Okon SU, Addey CI, Ebido CC, Opabunmi AO, Okeke ES, Chukwudozie KI (2022). Antibiotic Resistance in the aquatic environment: Analytical techniques and interactive impact of emerging contaminants. Environ Toxicol Pharmacol.

[5] de Kraker ME, Stewardson AJ, Harbarth S (2016). Will 10 million people die a year due to Antimicrobial Resistance by 2050?. PLoS Med.

[6] He P, Wu Y, Huang W, Wu X, Lv J, Liu P, Bu L, Bai Z, Chen S, Feng W, Yang Z (2020). Characteristics of and variation in airborne ARGs among urban hospitals and adjacent urban and suburban communities: a metagenomic approach. Environ Int.

[7] The Lancet Planetary H (2018). The natural environment and emergence of antibiotic resistance. Lancet Planet Health.

[8] Zhang S, Abbas M, Rehman MU, Huang Y, Zhou R, Gong S, Yang H, Chen S, Wang M, Cheng A (2020). Dissemination of antibiotic resistance genes (ARGs) via integrons in Escherichia coli: a risk to human health. Environ Pollut.

[9] Pal C, Bengtsson-Palme J, Kristiansson E, Larsson DG (2016). The structure and diversity of human, animal and environmental resistomes. Microbiome.

[10] Sommer MOA, Dantas G, Church GM (2009). Functional characterization of the antibiotic resistance reservoir in the human microflora. Science.

[11] Kim S, Covington A, Pamer EG (2017). The intestinal microbiota: antibiotics, colonization resistance, and enteric pathogens. Immunol Rev.

[12] Forsberg KJ, Patel S, Gibson MK, Lauber CL, Knight R, Fierer N, Dantas G (2014). Bacterial phylogeny structures soil resistomes across habitats. Nature.

[13] Pehrsson EC, Tsukayama P, Patel S, Mejia-Bautista M, Sosa-Soto G, Navarrete KM, Calderon M, Cabrera L, Hoyos-Arango W, Bertoli MT, Berg DE, Gilman RH, Dantas G (2016). Interconnected microbiomes and resistomes in low-income human habitats. Nature.

[14] Van Gompel L, Luiken REC, Hansen RB, Munk P, Bouwknegt M, Heres L, Greve GD, Scherpenisse P, Jongerius-Gortemaker BGM, Tersteeg-Zijderveld MHG, Garcia-Cobos S, Dohmen W, Dorado-Garcia A, Wagenaar JA, Urlings BAP, Aarestrup FM, Mevius DJ, Heederik DJJ, Schmitt H, Bossers A, Smit LAM (2020). Description and determinants of the faecal resistome and microbiome of farmers and slaughterhouse workers: a metagenome-wide cross-sectional study. Environ Int.

[15] Kaper JB, Nataro JP, Mobley HL (2004). Pathogenic Escherichia coli. Nat Rev Microbiol.

[16] Hossain A, Habibullah-Al-Mamun M, Nagano I, Masunaga S, Kitazawa D, Matsuda H (2022). Antibiotics, antibiotic-resistant bacteria, and resistance genes in aquaculture: risks, current concern, and future thinking. Environ Sci Pollut Res Int.

[17] Marshall BM, Levy SB (2011). Food animals and antimicrobials: impacts on human health. Clin Microbiol Rev.

[18] Zhang QQ, Ying GG, Pan CG, Liu YS, Zhao JL (2015). Comprehensive evaluation of antibiotics emission and fate in the river basins of China: source analysis, multimedia modeling, and linkage to bacterial resistance. Environ Sci Technol.

[19] Xiao L, Estelle J, Kiilerich P, Ramayo-Caldas Y, Xia Z, Feng Q, Liang S, Pedersen AO, Kjeldsen NJ, Liu C, Maguin E, Dore J, Pons N, Le Chatelier E, Prifti E, Li J, Jia H, Liu X, Xu X, Ehrlich SD, Madsen L, Kristiansen K, Rogel-Gaillard C, Wang J (2016). A reference gene catalogue of the pig gut microbiome. Nat Microbiol.

[20] Mu C, Yang Y, Su Y, Zoetendal EG, Zhu W (2017). Differences in Microbiota Membership along the gastrointestinal tract of piglets and their Differential alterations following an early-life antibiotic intervention. Front Microbiol.

[21] Wang Q, Duan YJ, Wang SP, Wang LT, Hou ZL, Cui YX, Hou J, Das R, Mao DQ, Luo Y (2020). Occurrence and distribution of clinical and veterinary antibiotics in the faeces of a Chinese population. J Hazard Mater.

[22] Alicea-Serrano AM, Contreras M, Magris M, Hidalgo G, Dominguez-Bello MG (2013). Tetracycline resistance genes acquired at birth. Arch Microbiol.

[23] Yassour M, Jason E, Hogstrom LJ, Arthur TD, Tripathi S, Siljander H, Selvenius J, Oikarinen S, Hyoty H, Virtanen SM, Ilonen J, Ferretti P, Pasolli E, Tett A, Asnicar F, Segata N, Vlamakis H, Lander ES, Huttenhower C, Knip M, Xavier RJ (2018). Strain-level analysis of Mother-to-child bacterial transmission during the First few months of life. Cell Host Microbe.

[24] Yang Y, Chen N, Sun L, Zhang Y, Wu Y, Wang Y, Liao X, Mi J (2021). Short-term cold stress can reduce the abundance of antibiotic resistance genes in the cecum and feces in a pig model. J Hazard Mater.

[25] Zhang AN, Gaston JM, Dai CL, Zhao S, Poyet M, Groussin M, Yin X, Li LG, van Loosdrecht MCM, Topp E, Gillings MR, Hanage WP, Tiedje JM, Moniz K, Alm EJ, Zhang T (2021). An omics-based framework for assessing the health risk of antimicrobial resistance genes. Nat Commun.

[26] Luiken REC, Van Gompel L, Bossers A, Munk P, Joosten P, Hansen RB, Knudsen BE, Garcia-Cobos S, Dewulf J, Aarestrup FM, Wagenaar JA, Smit LAM, Mevius DJ, Heederik DJJ, Schmitt H, group E (2020). Farm dust resistomes and bacterial microbiomes in European poultry and pig farms. Environ Int.

[27] Liu J, Taft DH, Maldonado-Gomez MX, Johnson D, Treiber ML, Lemay DG, DePeters EJ, Mills DA (2019). The fecal resistome of dairy cattle is associated with diet during nursing. Nat Commun.

[28] Adam HJ, Baxter MR, Davidson RJ, Rubinstein E, Fanella S, Karlowsky JA, Lagace-Wiens PR, Hoban DJ, Zhanel GG, Canadian Antimicrobial Resistance A (2013). Comparison of pathogens and their antimicrobial resistance patterns in paediatric, adult and elderly patients in Canadian hospitals. J Antimicrob Chemother.

[29] Toya R, Sasaki Y, Uemura R, Sueyoshi M (2021). Indications and patterns of antimicrobial use in pig farms in the southern Kyushu, Japan: large amounts of tetracyclines used to treat Respiratory Disease in post-weaning and fattening pigs. J Vet Med Sci.

[30] van Rennings L, von Munchhausen C, Ottilie H, Hartmann M, Merle R, Honscha W, Kasbohrer A, Kreienbrock L (2015). Cross-sectional study on antibiotic usage in pigs in Germany. PLoS ONE.

[31] Trauffler M, Griesbacher A, Fuchs K, Kofer J (2014). Antimicrobial drug use in Austrian pig farms: plausibility check of electronic on-farm records and estimation of consumption. Vet Rec.

[32] Jensen VF, Emborg HD, Aarestrup FM (2012). Indications and patterns of therapeutic use of antimicrobial agents in the Danish pig production from 2002 to 2008. J Vet Pharmacol Ther.

[33] Duan Y, Chen Z, Tan L, Wang X, Xue Y, Wang S, Wang Q, Das R, Lin H, Hou J, Li L, Mao D, Luo Y (2020). Gut resistomes, microbiota and antibiotic residues in Chinese patients undergoing antibiotic administration and healthy individuals. Sci Total Environ.

[34] Zhou ZC, Liu Y, Lin ZJ, Shuai XY, Zhu L, Xu L, Meng LX, Sun YJ, Chen H (2021). Spread of antibiotic resistance genes and microbiota in airborne particulate matter, dust, and human airways in the urban hospital. Environ Int.

[35] Ma L, Xia Y, Li B, Yang Y, Li LG, Tiedje JM, Zhang T (2016). Metagenomic Assembly reveals hosts of Antibiotic Resistance genes and the Shared Resistome in Pig, Chicken, and human feces. Environ Sci Technol.

[36] Spellberg B, Doi Y (2015). The rise of fluoroquinolone-resistant Escherichia coli in the community: scarier than we thought. J Infect Dis.

[37] Mather AE, Baker KS, McGregor H, Coupland P, Mather PL, Deheer-Graham A, Parkhill J, Bracegirdle P, Russell JE, Thomson NR (2014). Bacillary Dysentery from World War 1 and NCTC1, the first bacterial isolate in the National Collection. Lancet.

[38] Galimand M, Fishovitz J, Lambert T, Barbe V, Zajicek J, Mobashery S, Courvalin P (2015). AAC(3)-XI, a new aminoglycoside 3-N-acetyltransferase from Corynebacterium striatum. Antimicrob Agents Chemother.

[39] Martinez JL, Coque TM, Baquero F (2015). What is a resistance gene? Ranking risk in resistomes. Nat Rev Microbiol.

[40] Zhang Z, Zhang Q, Wang T, Xu N, Lu T, Hong W, Penuelas J, Gillings M, Wang M, Gao W, Qian H (2022). Assessment of global health risk of antibiotic resistance genes. Nat Commun.

[41] Bolger AM, Lohse M, Usadel B (2014). Trimmomatic: a flexible trimmer for Illumina sequence data. Bioinformatics.

[42] Yang Y, Jiang X, Chai B, Ma L, Li B, Zhang A, Cole JR, Tiedje JM, Zhang T (2016). ARGs-OAP: online analysis pipeline for antibiotic resistance genes detection from metagenomic data using an integrated structured ARG-database. Bioinformatics.

[43] Yin X, Jiang XT, Chai B, Li L, Yang Y, Cole JR, Tiedje JM, Zhang T (2018). ARGs-OAP v2.0 with an expanded SARG database and hidden Markov models for enhancement characterization and quantification of antibiotic resistance genes in environmental metagenomes. Bioinformatics.

[44] Parnanen K, Karkman A, Hultman J, Lyra C, Bengtsson-Palme J, Larsson DGJ, Rautava S, Isolauri E, Salminen S, Kumar H, Satokari R, Virta M (2018). Maternal gut and breast milk microbiota affect infant gut antibiotic resistome and mobile genetic elements. Nat Commun.

[45] Valenzuela-Gonzalez F, Martinez-Porchas M, Villalpando-Canchola E, Vargas-Albores F (2016). Studying long 16S rDNA sequences with ultrafast-metagenomic sequence classification using exact alignments (Kraken). J Microbiol Methods.

[46] McArdle AJ, Kaforou M (2020). Sensitivity of shotgun metagenomics to host DNA: abundance estimates depend on bioinformatic tools and contamination is the main issue. Access Microbiol.

[47] Su G, Morris JH, Demchak B, Bader GD (2014). Biological network exploration with Cytoscape 3. Curr Protoc Bioinformatics.

