# Supplementary material for: Longitudinal metagenomic study reveals the dynamics of fecal antibiotic resistome in pigs throughout the lifetime
Source: Anim Microbiome. 2023 Nov 8;5:55. doi: 10.1186/s42523-023-00279-z (PMC10634126; doi:10.1186/s42523-023-00279-z)
Supplement: Supplementary file 1 — Supplementary Material 1 [file 42523_2023_279_MOESM1_ESM.docx]

**Longitudinal metagenomic study reveals the dynamics of fecal antibiotic resistome in pigs throughout the lifetime**

**Supplementary Table 1-5**

**Table S1** Statistical analysis results for the Fig.1A, B and Fig.3B.

**Table S2** Procrustes analysis to correlate the association between the microbial community, ARGs and MGEs.

**Table S3** Description of the 162 sampling sites.

**Table S4** Abundance of antibiotic resistant gene types (normalized by 16s RNA).

**Table S5** The total antibiotic resistant gene subtypes (normalized by 16s RNA).

**Table S6** Abundance of mobile genetic elements (normalized by 16s RNA).

**Table S7** The total mobile genetic elements subtypes (normalized by 16s RNA).

**Note：**Table S1-S5 are individual excel files (.xlsx)

**Table S2**. Procrustes analysis to correlate the association between the microbial community, ARGs and MGEs.

| **Comparison** | **Mantel_r** | **Mantel_p** | **Proc_r** | **Proc_p** |
| --- | --- | --- | --- | --- |
| ARG vs Microbe | 0.736 | 0.001 | 0.387 | 0.001 |
| ARG vs MRG | 0.756 | 0.001 | 0.440 | 0.001 |
| ARG vs MGE | 0.539 | 0.001 | 0.710 | 0.001 |
| Microbe vs MRG | 0.663 | 0.001 | 0.671 | 0.001 |
| Microbe vs MGE | 0.394 | 0.001 | 0.748 | 0.001 |
| MRG vs MGE | 0.625 | 0.001 | 0.683 | 0.001 |

**Supplementary Figure 1-6**

**Figure S1** Sampling site in Lin’an, Hangzhou, Zhejiang province, China.

**Figure S2** Principal coordinates analysis (PCoA) using Bray-Curtis distances based on abundance of ARGs revealed a significant difference ARGs patten between pig samples.

**Figure S3** Different resistance mechanisms among the different growth stage in pigs.

**Figure S4** The co-occurrence patterns among ARG subtypes.

**Figure S5** Microbiome profiles among the different growth stage in pigs.

**Figure S6** Characteristics of MGEs among the different growth stage in pigs.

**Figure S1**


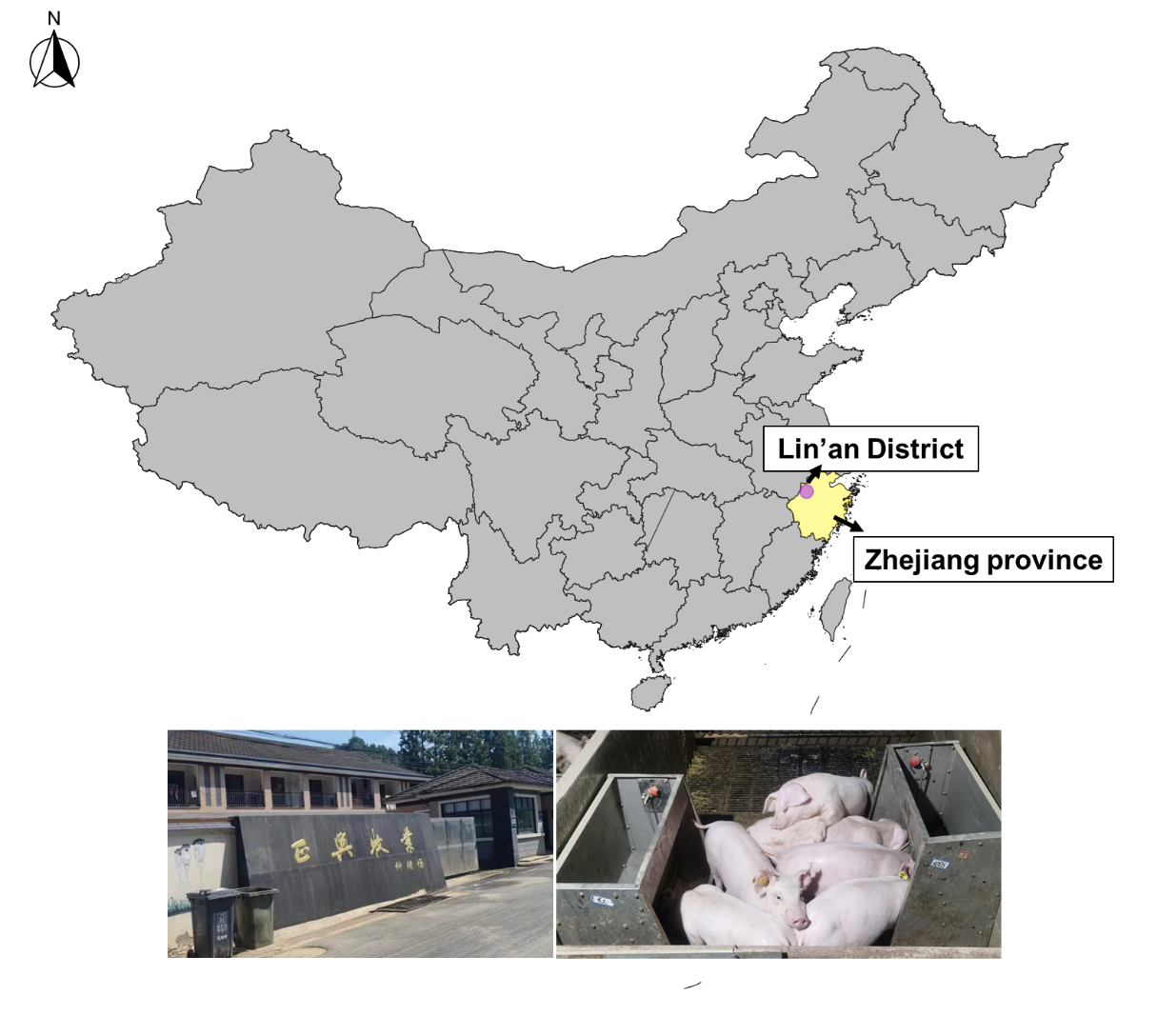


**Fig.S1.** Sampling site in Lin’an, Hangzhou, Zhejiang province, China.

**Figure S2**


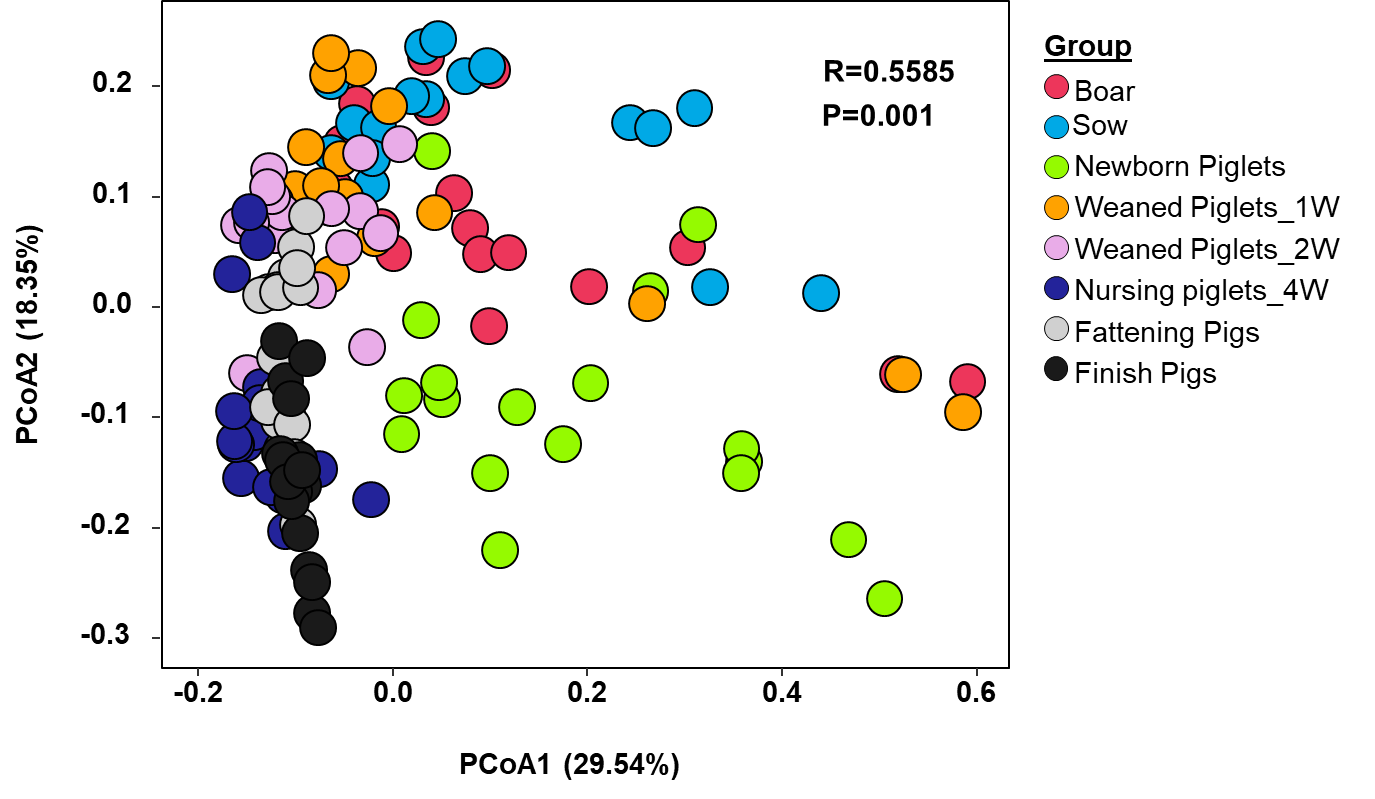


**Fig.S2.** Principal coordinates analysis (PCoA) using Bray-Curtis distances based on abundance of ARGs revealed a significant difference ARGs patten between pig samples.

**Figure S3**

**
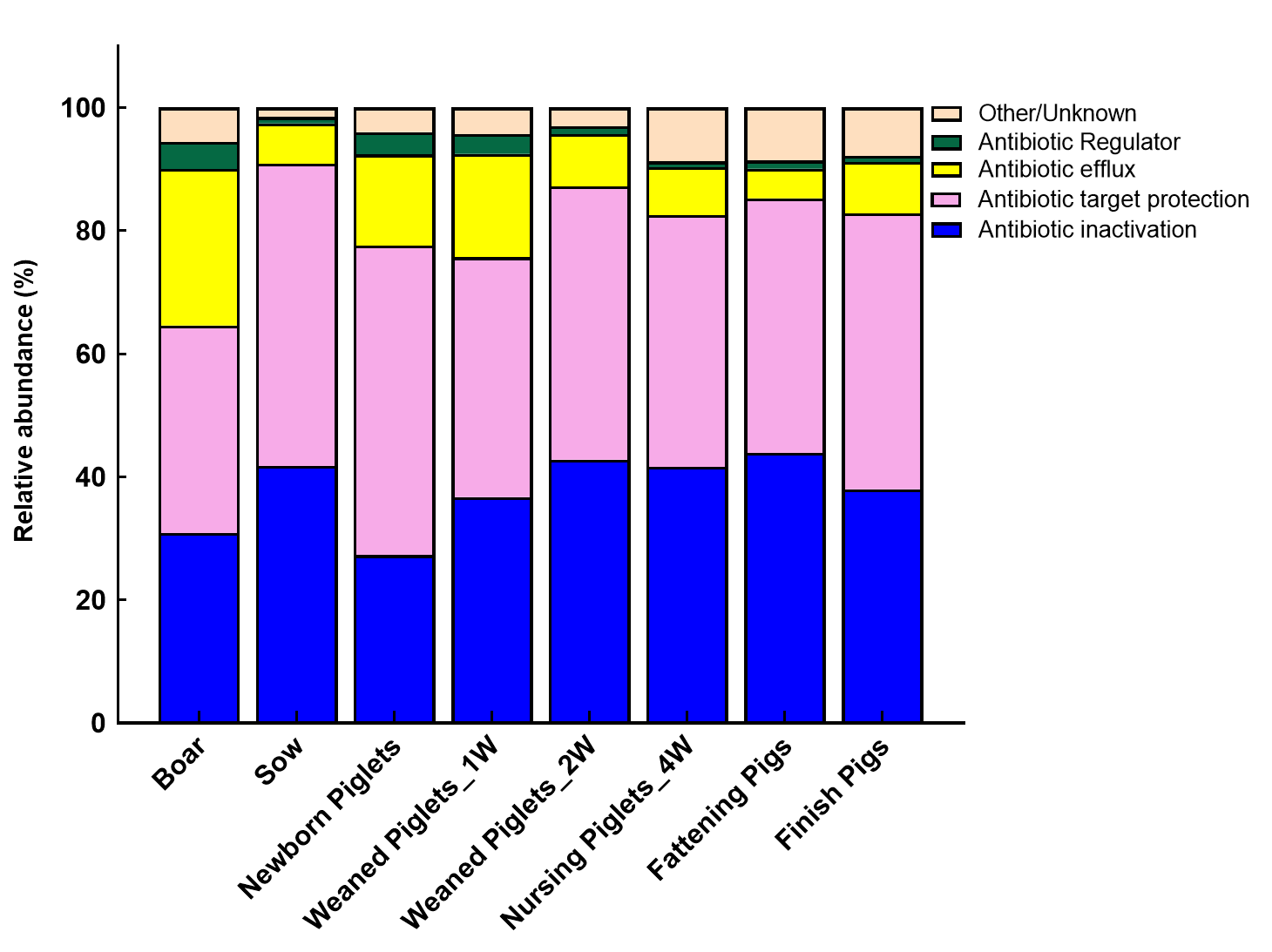
**

**Fig.S3.** Different resistance mechanisms among the different growth stage in pigs.

**Figure S4**

**
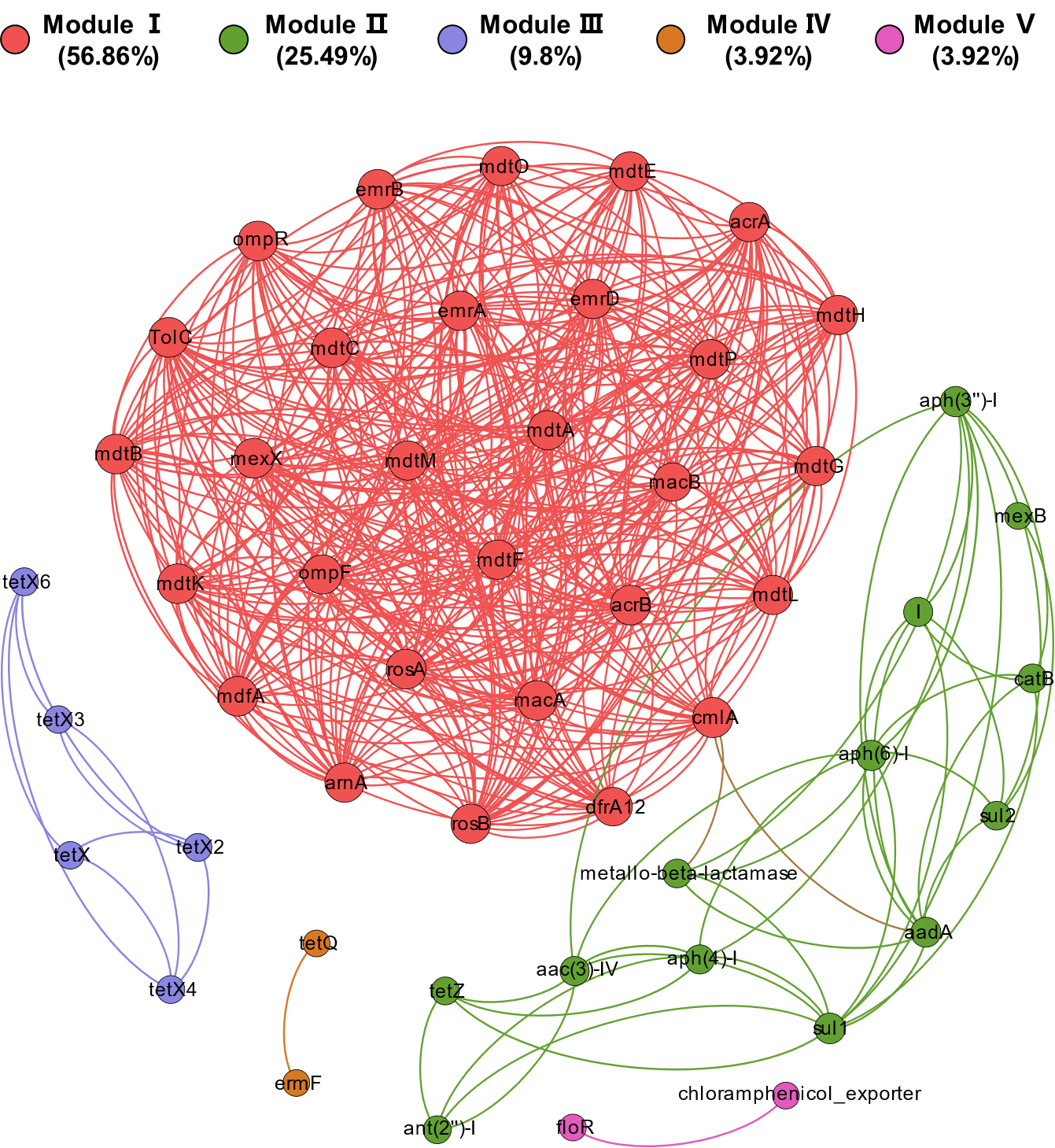
**

**Fig.S4.** The co-occurrence patterns among ARG subtypes were explored using network inference based on strong (Spearman’s correlation coefficient (ρ)>0.9) and significant (*p*-value <0.01) correlations.

**Figure S5**


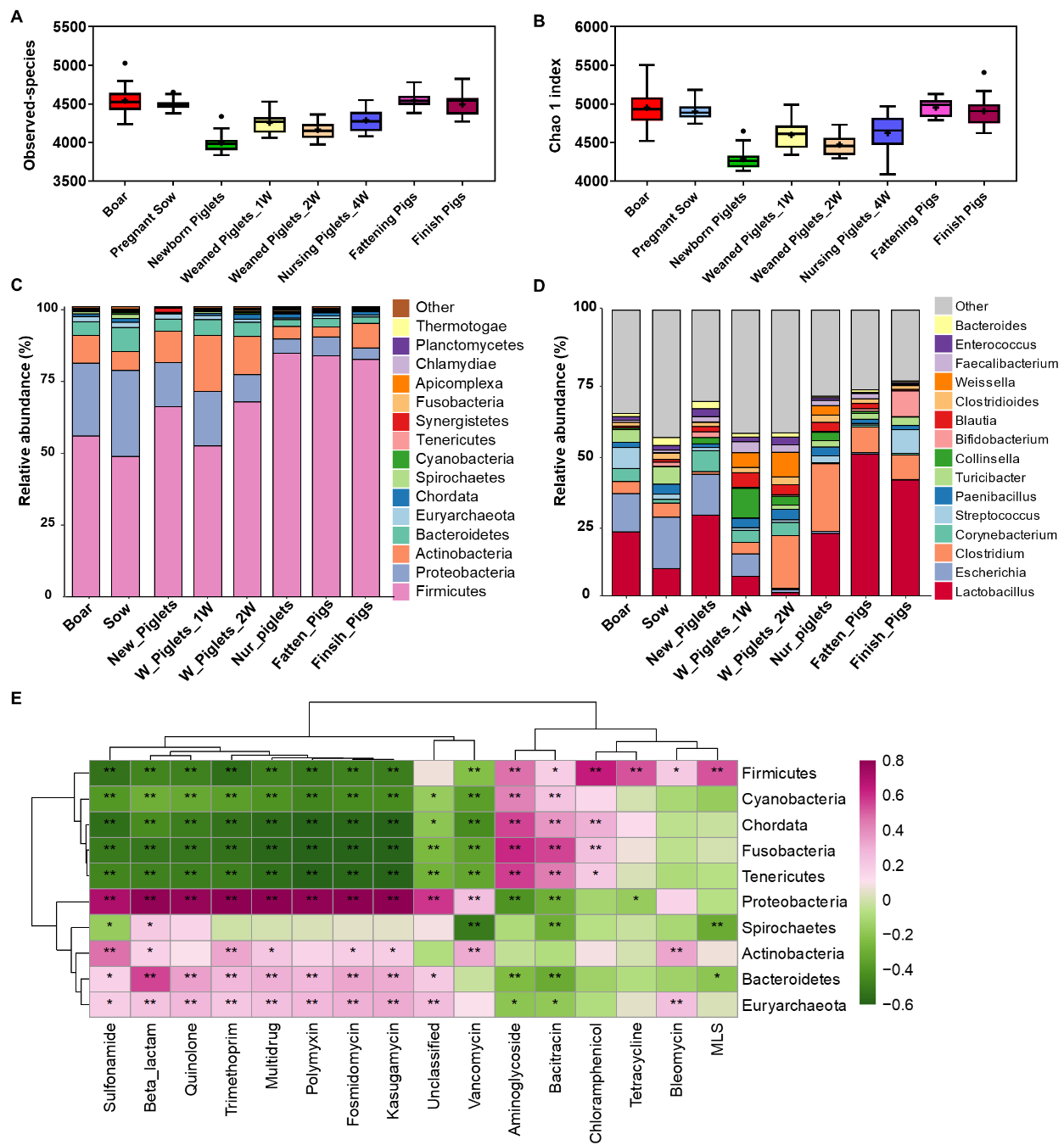


**Fig.S5.** Microbiome profiles among the different growth stage in pigs**.** (A) Observed species among groups. (B) Chao 1 index. Relative abundance in phylum (C) and genus levels (D). (E) The correlation analysis between bacteria (phylum levels) and ARGs types (Spearman’s correlation coefficient (ρ)>0.6) and significant (*p*-value <0.01)).

**Figure S6**


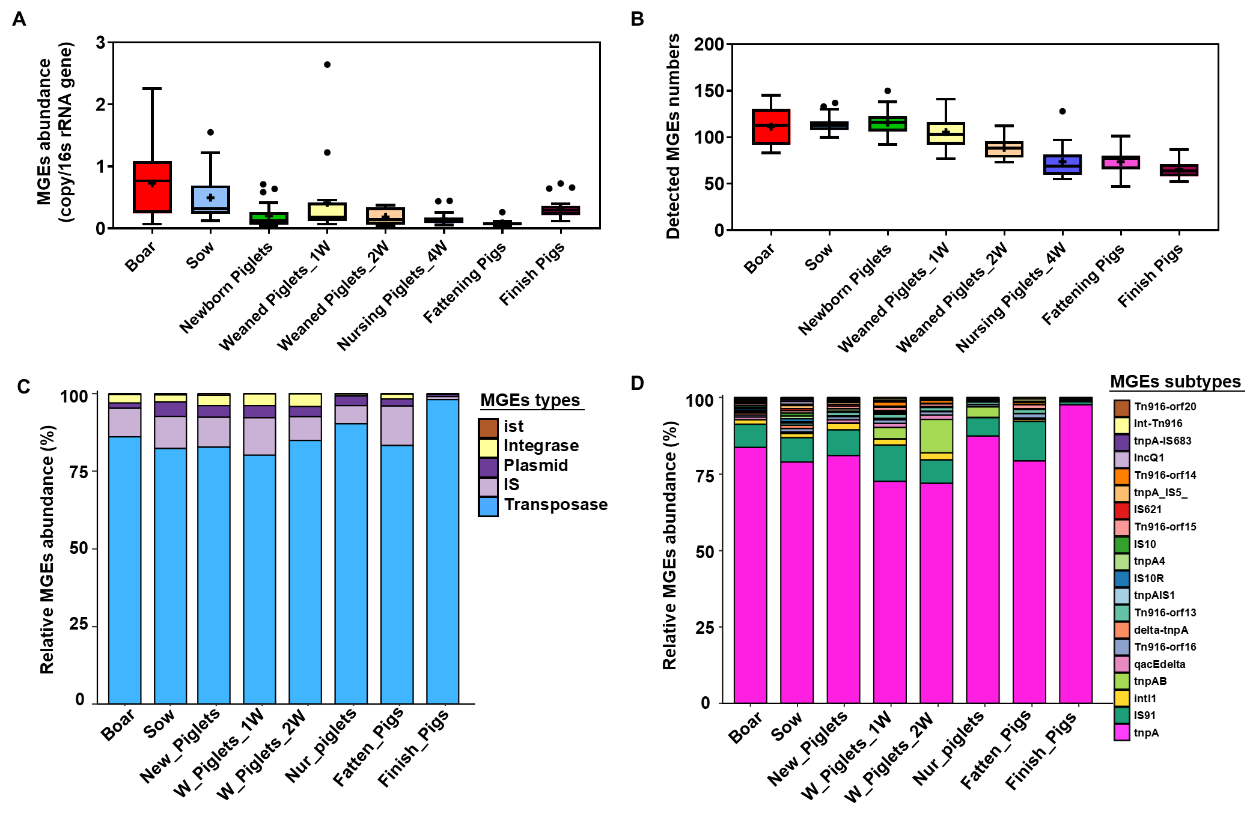


**Fig.S6.** Characteristics of MGEs among the different growth stage in pigs. (A) Box plot showing the total MGEs type abundance (copy/16s rRNA gene) in each group. (B) Detected ARG subtypes numbers of each group. (C) Distribution of relative MGEs abundance in pigs with different types. (D) Top 20 MGEs subtypes based on the abundance in each sample.
